# Synthesis and Bioactivities of Marine Pyran-Isoindolone Derivatives as Potential Antithrombotic Agents

**DOI:** 10.3390/md19040218

**Published:** 2021-04-15

**Authors:** Yinan Wang, Hui Chen, Ruilong Sheng, Zhe Fu, Junting Fan, Wenhui Wu, Qidong Tu, Ruihua Guo

**Affiliations:** 1 College of Food Science and Technology, Shanghai Ocean University, Shanghai 201306, China; wynshou@163.com (Y.W.); fzshou2019@163.com (Z.F.); whwu@shou.edu.cn (W.W.); 2 Shanghai Engineering Research Center of Hadal Science and Technology, College of Marine Sciences, Shanghai Ocean University, Shanghai 201306, China; h-chen@shou.edu.cn; 3 CQM-Centro de Química da Madeira, Campus da Penteada, Universidade da Madeira, 9000-390 Funchal, Portugal; ruilong.sheng@staff.uma.pt; 4 School of Pharmacy, Jiangxi Science and Technology Normal University, Nanchang 330013, China; 5 School of Pharmacy, Nanjing Medical University, Nanjing 211166, China; juntingfan@njmu.edu.cn; 6 Shanghai Engineering Research Center of Aquatic-Product Processing & Preservation, Shanghai 201306, China; 7 Laboratory of Quality and Safety Risk Assessment for Aquatic Products on Storage and Preservation (Shanghai), Ministry of Agriculture, Shanghai 201306, China

**Keywords:** pyran-isoindolone derivatives, antithrombotic agents, fibrinolytic activity, Pro-uPA-catalyzed plasminogen, modification

## Abstract

2,5-Bis-[8-(4,8-dimethyl-nona-3,7-dienyl)-5,7-dihydroxy-8-methyl-3-keto-1,2,7,8-teraahydro-6*H*-pyran[*a*]isoindol-2-yl]-pentanoic acid (FGFC1) is a marine pyran-isoindolone derivative isolated from a rare marine microorganism *Stachybotrys longispora* FG216, which showed moderate antithrombotic(fibrinolytic) activity. To further enhance its antithrombotic effect, a series of new FGFC1 derivatives (**F1**–**F7**) were synthesized via chemical modification at C-2 and C-2′ phenol groups moieties and C-1″ carboxyl group. Their fibrinolytic activities in vitro were evaluated. Among the derivatives, **F1**–**F4** and **F6** showed significant fibrinolytic activities with EC_50_ of 59.7, 87.1, 66.6, 82.8, and 42.3 μM, respectively, via enhancement of urokinase activity. Notably, derivative **F6** presented the most remarkable fibrinolytic activity (2.72-fold than that of FGFC1). Furthermore, the cytotoxicity of derivative **F6** was tested as well as expression of Fas/Apo-1 and IL-1 on HeLa cells. The results showed that, compared to FGFC1, derivative **F6** possessed moderate cytotoxicity and apoptotic effect on HeLa cells (statistical significance *p* > 0.1), making **F6** a potential antithrombotic agent towards clinical application.

## 1. Introduction

Nowadays, non-transmissible chronic diseases have become fundamental medical problems [1] including cardiovascular, neurological, rheumatologic, diabetic, etc. Among them, cardiovascular disease is one leading cause of death in non-transmissible disease. The World Health Organization reported that approximately 18 million people died from cardiovascular disease each year [2]. Moreover, cardiovascular disease originated mostly from thrombosis [3], such as arterial and venous thrombosis, atherosclerosis, heart attacks, strokes and peripheral vascular diseases, whose death rate was close to that of cancer in recent years [4]. It is estimated that more than 1 million people die from cardiovascular disease every year in the United States [5]. Due to genetics and environmental influence, thrombosis has become recurrent and the age of onset has become younger [6], which seriously threaten human beings’ health worldwide [7,8].

To date, there are three generations of drugs that have been used for the treatments of thrombosis. The first generation of thrombolytic agent is an antigenic bacterial product with non-specificity and thrombolytic activities, including streptokinase (SK) [9] and urokinase (UK) [10]. They can transform plasminogen into active plasmin, which is combined with fibrin for promoting the dissolution of the thrombus. However, they result in bleeding diathesis [11]. The second-generation features improved fibrin specificity, containing tissue plasminogen activator (t-PA) [12], single-chain urokinase plasminogen activator (rscu-PA) [13] and acetylated plasminogen streptokinase activator complex (APSAC) [14]. The drugs can activate fibrin-associated plasminogen, causing plasminogen or α2-antiplasmindepletion or systemic fibrinogen disintegration. Nevertheless, it is limited due to its short half-life and serious anaphylactic reaction [15]. The third generation can lengthen half-life, increase resistance to plasma protease inhibitors, and bind to fibrin, more selectively including TNK-PA [16], STAR, r-PA [17], etc. [18]. However, they also have the drawback of bleeding risks [19]. Therefore, the exploration of small-molecule drugs with antithrombotic activities has received increasing attention owing to their great potential and security in the safety of thrombus. Currently, many small-molecule antithrombotic agents have been reported, including warfarin, apixaban, edoxaba, rivaroxaban, dabigatran, the vitamin K antagonists, etc. [20,21,22,23].

At present, the discovery of most drugs originates from chemical modifications of natural products [24,25,26,27,28,29]. Thus, chemists are devoted to synthesizing various new compounds using diverse natural products as substrates. Moreover, they found that those compounds have better biological activity than natural products [30,31].

**Scheme 1:**
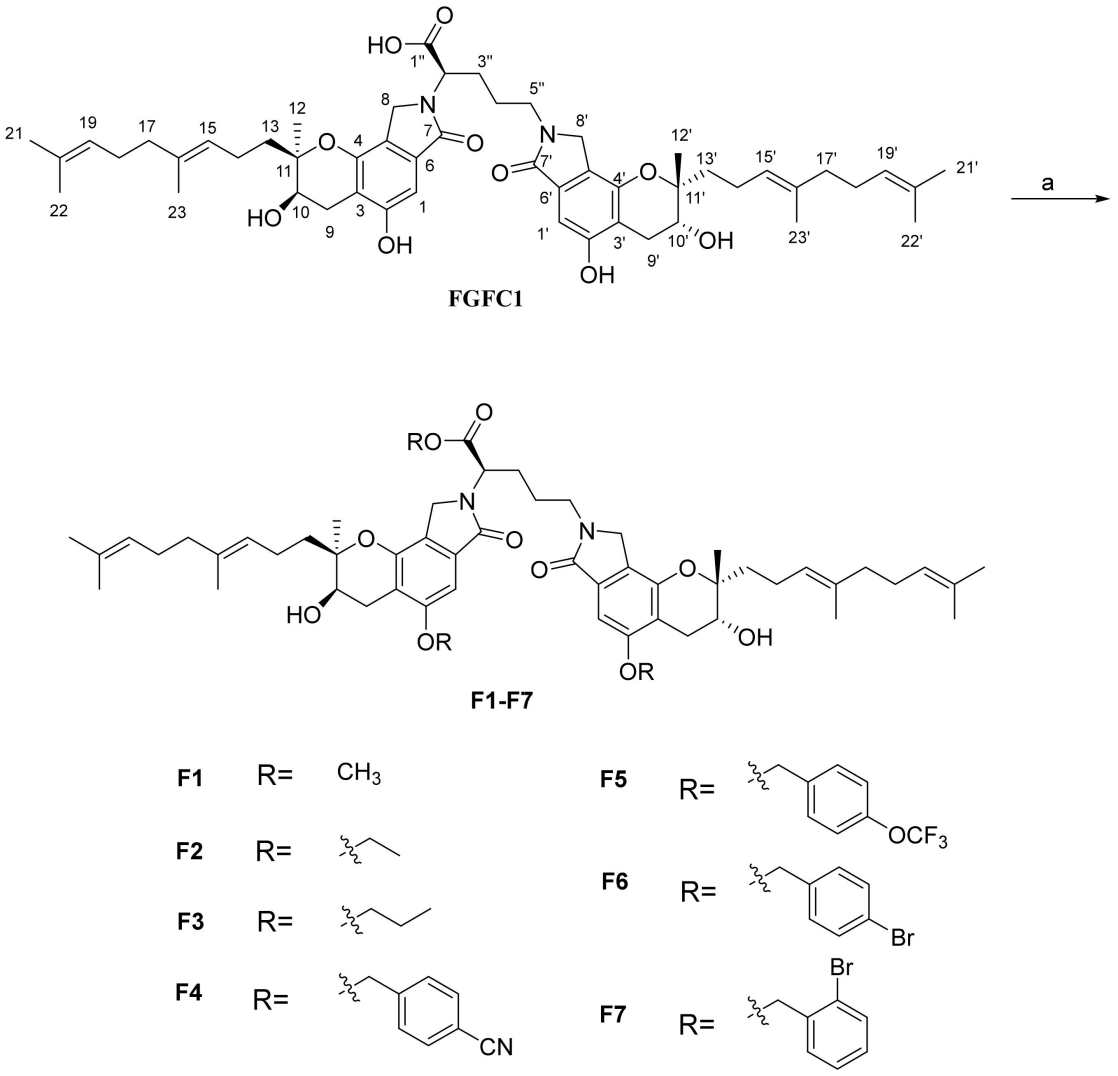
Synthesis of compounds **F1**–**F7**. Reagents and conditions: (a) RX (X = I, Br), K_2_CO_3_, acetone or *N*,*N*-dimethylacetamide, reflux, 2–3 h, yield 50–75%.

Previously, a marine pyran-isoindolone derivative, 2,5-bis-[8-(4,8-dimethyl-nona-3,7-dienyl)-5,7-dihydroxy-8-methyl-3-keto-1,2,7,8-tetrahydro-6H-pyran [a]isoindol-2-yl]-pentanoic acid (FGFC1, Scheme 1), was isolated from a rare marine microorganism Stachybotrys longispora FG216 (CCTCC No M2012272) in our laboratory [32]. In our previous report, FGFC1 exhibited fibrinolytic activity in vitro and in vivo [33]. However, fibrinolytic activity and stability of FGFC1 have limited its further investigation as a thrombolytic candidate for therapeutic effects [34,35,36,37]. Phenolic hydroxyl moieties (C2-OH/C2′-OH) and carboxyl fragment (C1″-COOH) are important functional groups in FGFC1. Our previous works showed that liphatic and benzyl derivatives possessed significant potency [38,39]. Therefore, pyran-isoindolone derivatives **F1**–**F7** were synthesized using compound FGFC1 as a starting material via chemical modification at C-2 and C-2′ phenol groups moieties and C-1″ carboxyl group (Scheme 1). Furthermore, their fibrinolytic activities were also evaluated. The results indicated that **F6** possessed the most remarkable fibrinolytic activity. Then, its cytotoxicity was tested as well as Fas/Apo-1 and IL-1 on HeLa cells with satisfactory results.

## 2. Results and Discussion

### 2.1. Chemistry

FGFC1 had two types of acidic functional groups: phenolic phenol groups moieties (C2-OH/C2′-OH) and carboxyl fragment (C1″-COOH). Whether FGFC1 was oral or injected, its acidic groups would cause unnecessary irritation at the site of administration. Thus, the acidity of FGFC1 should be alleviated but its stable antithrombotic activity needs to be maintained, which could be completed through chemical modification including esterification and etherification. Nucleophilic oxygen anions at C-2, C2′ and C-1″ in FGFC1 reacted with various substituted halo-(iodo or bromo) hydrocarbon compounds in the presence of K_2_CO_3_ to prepare new FGFC1 derivatives, with phenolic ether groups at C-2 and C2′, and aliphatic ester groups at C-1″. The hydrophobic modification reaction of FGFC1 performed on C-2 and C-2′ phenol groups moieties and C-1″carboxyl group is shown in Scheme 1. FGFC1 was treated with iodomethane, bromoethane, and bromopropane in the presence of K_2_CO_3_ in acetone to yield derivatives **F1**–**F3,** with the isolated yields ranging from 62% to 75%. Moreover, FGFC1 was treated with 4-bromobenzonitrile, 4-(trifluoromethoxy)benzyl bromide, 4-bromobenzyl bromide, and 2-bromobenzyl bromide in the presence of K_2_CO_3_ in *N*,*N*-dimethylacetamide to yield derivatives **F4**–**F7,** with the isolated yields ranging from 50% to 70%. Finally, molecular structures of the synthesized FGFC1 derivatives (**F1**–**F7**) were fully characterized in the Supplementary Materials.

### 2.2. Fibrinolytic Activities of FGFC1 and F1–F7 In Vitro

**Figure 1:**
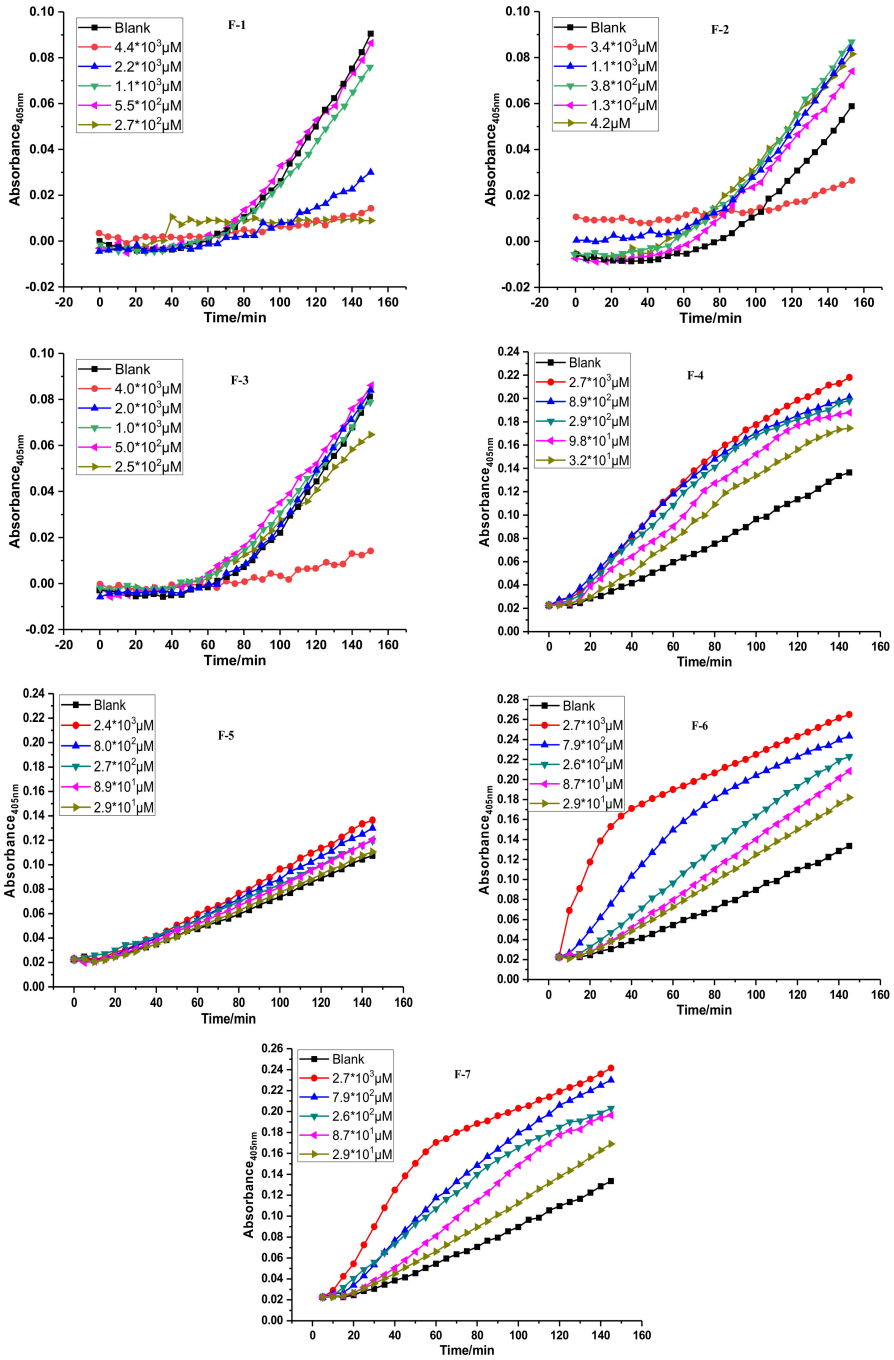
The effect of derivatives **F1**–**F7** on the fibrinolytic activities of the reciprocal activation of Pro-uPA-catalyzed plasminogen.

**Figure 2:**
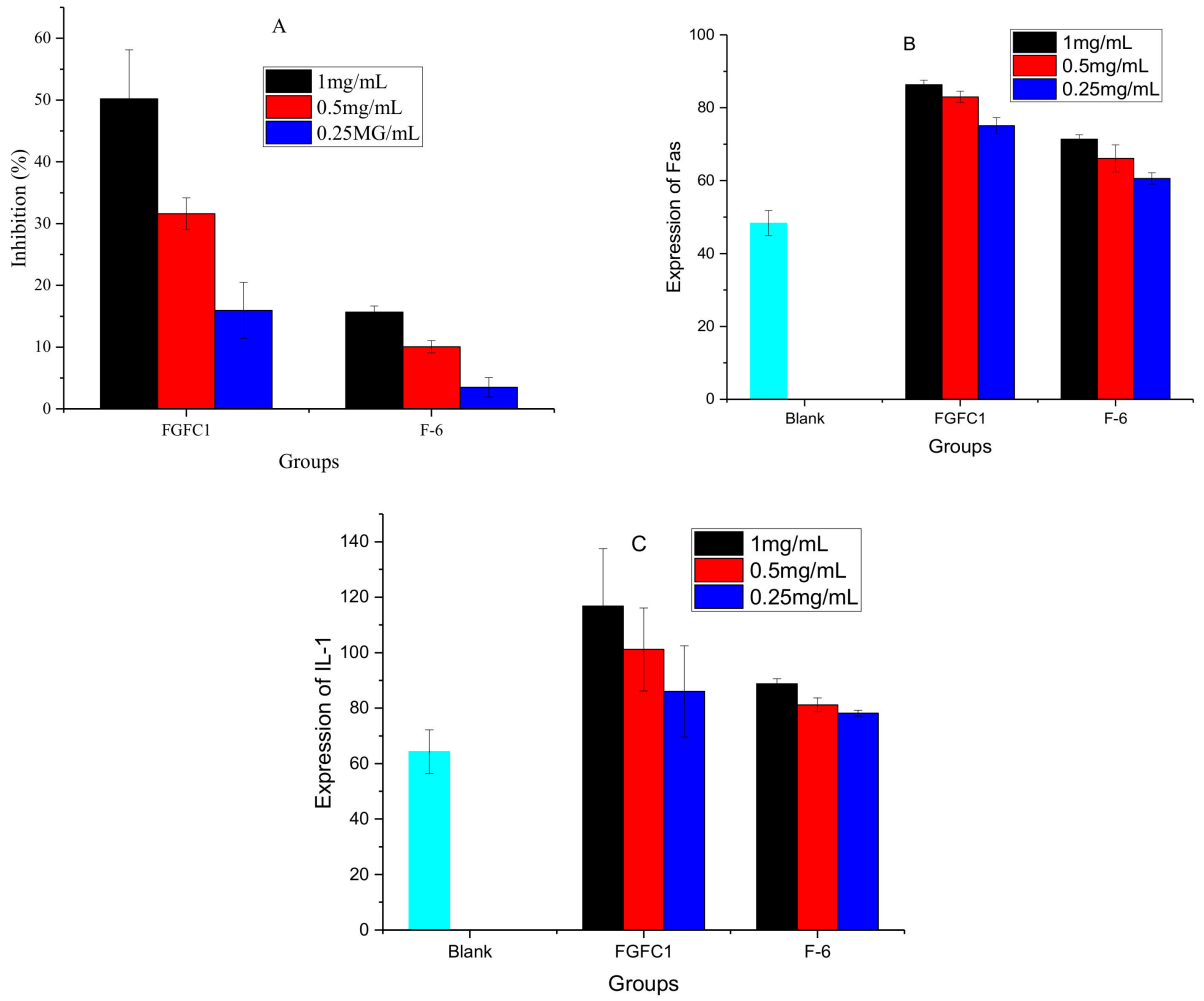
The biological effect of **F6** on HeLa cells, cytotoxicity (**A**), expression of Fas/Apo-1 (**B**), and expression of IL-1 (**C**) were determined in plasma, with FGFC1 as the reference.

To evaluate the antithrombotic effect, all the synthesized FGFC1 derivatives were tested for their fibrinolytic activities in vitro. Fibrinolytic activity of each compound was expressed as 50% effective concentration (EC_50_), and FGFC1 was used as a positive control. The reciprocal activation of pro-uPA catalyzed plasminogen, which was measured based on urokinase activity by a chromogenic substrate S-2444. EC_50_ was calculated with the slope of kinetic curve of enzymatic reaction based on the reciprocal activation of pro-uPA and plasminogen (Figure 1 and Figure 2) [40]. The results are shown in Table 1. The effect of introducing different hydrophobic moieties/groups to C-2, C-2′ and C-1′′ positions of FGFC1 was investigated by replacement of the protons on C2-OH/C2′-OH moieties and C1′′-COOH position, with methyl, ethyl, propyl, and bromo-substituted benzyl halide groups to yield derivatives **F1**–**F7** (purity > 98% by HPLC analysis), which presented different urokinase activities and reciprocal activation of Pro-uPA-catalyzed plasminogen in vitro.

The synthesized FGFC1 derivatives included aliphatic **F1**–**F3** and benzyl **F4**–**F7** compounds. For derivatives **F1**–**F3**, aliphatic (methyl, ethyl and *n*-propyl) groups were introduced to C-2, C-2′ and C-1″ positions in FGFC1. The EC_50_ values showed that the fibrinolytic activity of derivatives **F1**–**F3** had a tendency as follows: with the increasing of aliphatic chain length, the EC_50_ gliding down initially from 59.7 μM (**F1**) to 87.1 μM (**F2**) and then climbing slowly to 66.6 μM (**F3**). Interestingly, all of the aliphatic derivatives **F1**–**F3** were more active (1.3–2.0 fold) than FGFC1. This result suggested that substituted C-2, C-2′ and C-1″ alkyl moieties could confer increased fibrinolytic activity. Methyl-substituted derivatives **F1** had the most potent fibrinolytic activity with EC_50_ value of 59.7 μM. However, the kinetic curves of derivatives **F1**–**F3** (Figure 2) indicated the fibrinolytic activity in vitro was not an absolutely dose-dependent. Especially at high concentration (4.0 mg/mL), it presented a relatively flat trend, indicating the unsatisfactory performance of aliphatic substituted derivatives **F1**–**F3** at 4.0 mg/mL.

For benzyl derivatives **F4**–**F7**, **F4** (EC_50_ = 82.8 μM) and **F6** (EC_50_ = 42.3 μM) at the 4-position of benzyl scaffold were beneficial to fibrinolytic activity. 4-Br-containing **F6** possessed excellent fibrinolytic activity with an EC_50_ value of 42.3 μM (2.72-fold than FGFC1). Derivative **F5**, where a trifluoromethoxy group was introduced into the *para* position of benzene ring, markedly decreased the fibrinolytic activity. Shifting Br atom from *para* to ortho position to produce 3-Br-containing **F7** resulted in retained fibrinolytic activity (EC_50_ = 119.6 μM for **F7** vs. EC_50_ = 115.0 μM for FGFC1). The remarkably different EC_50_ of **F6** (42.3 μM) and **F7** (119.6 μM) implied that the bromo groups at *para* and *ortho* positions may bring them different urokinase-activating efficiencies. The kinetic curves of derivatives **F1**–**F7** showed that benzyl derivatives **F4**–**F7** possessed a dose- and time-dependent manner (Figure 1) and better fibrinolytic activity than aliphatic derivatives **F1**–**F3**. The curves of aliphatic derivatives **F1**–**F3** were concave functions; moreover, no distinct fluctuation was observed in the first 60 min. However, those of benzyl derivatives **F4**–**F7** were convex, with a rapid upward trend in the same span of time. These results showed that, by introducing aromatic rings, the fibrinolytic activity could be increased rapidly in the early stage and the fibrinolytic activity could be adjusted by changing the substitution groups, especially on **F6** and **F7**. Notably, **F6** possessed more effective and faster fibrinolytic activity than FGFC1, which was worthy of in-depth research and exploration.

### 2.3. Cytotoxicity, Expression of Fas/Apo-1 and IL-1 of F6 on HeLa Cells

The cytotoxicity of FGFC1 and derivative **F6** on HeLa cell lines was screened by MTT assay [41,42]. Figure 2A showed that FGFC1 and derivative **F6** possessed moderate cytotoxicity on HeLa cell lines. The introduction of substituted benzyl halide to the C-2, C-2′ and C-1″ position of FGFC1 decreased cytotoxicity, which increased with increasing concentration (0.25, 0.5, 1.0 mg/mL). Moreover, to evaluate the possible apoptosis of HeLa cells induced by **F6**, Fas/APO-1 (a type of cell-surface NGF/TNF receptor in cancer cell) assay was carried out (Figure 2B) [43,44,45]. To evaluate the immunogenic (inflammation) property, interleukin (IL-1) levels of derivative **F6** was also tested by the IL-1 assays (Figure 2C) [46]. The expression level of Fas was remarkably higher than that of the blank, but it was less than that of FGFC1 (Figure 2B). This meant that the ability of F6 to induce apoptosis was weaker than that of FGFC1. The expression level of IL-1 was obviously higher than that of blank, but it was less than that of FGFC1 (Figure 2C). This suggested that the inflammatory response of F6 was weaker than that of FGFC1. According to Figure 2B and Figure 2C, the negative effect of **F6** on tissue cell was weaker than that of FGFC1.

## 3. Experiment

### 3.1. Materials

All chemicals were analytical grade. Reagents and materials were obtained from commercial suppliers and used without further purification. FGFC1 was isolated from *S. longispora* FG216. Silica gel (200–300 mesh) for column chromatography was purchased from Qingdao Makall Group Co., Ltd. Pro-uPA, BSA (bovine serum albumin), plasminogen and plasmin were purchased from Sigma Aldrich (China). The chromogenic pyro-glutamyl-glycyl-l-arginine-p-nitroanilide S-2444 was purchased from BioMed. Tris-HCl buffer (100 mmol/L, NaCl, pH 7.4) and an enzyme-labeled (microplate reader) instrument (SH-1000, CORONA, Ibarakiken, Japan) were used throughout the fibrinolytic activity in vitro. Fas/Apo-1 Elisa kit and IL-1 Elisa kit were purchased from Shanghai Fusheng Industrial Co., Ltd. (China). Human cervical cancer cells (HeLa) were purchased from Shanghai Cell Bank of Chinese Academy of Sciences.

### 3.2. Chemistry

The syntheses of the derivatives were described in Supplementary Materials. Column chromatography (CC): silica gel (200–300 mesh; Qingdao Makall Group Co., Ltd.; Qingdao, China). All reactions were monitored using thin-layer chromatography (TLC) on silica gel plates.

Nuclear magnetic resonance spectra were recorded on a Bruker DRX 500 MHz NMR spectrometer. Mass spectra (MS) were recorded on an Advantage Max LCQ Thermo-Finnigan mass spectrometer. General procedure for preparation and original spectra of derivatives **F1**–**F7** are available in Supporting Information.

### 3.3. Fibrinolytic Activity in Vitro

The fibrinolytic activities of compounds FGFC1 and derivatives **F1**–**F7** were evaluated by plasmin method. Tris-HCl buffer solution (100 mmol/L, NaCl, pH 7.4), plasminogen (plg), BSA, and S-2444 were used as substrates. Derivatives **F1**–**F7** dissolved in 0.05 mol/L Tris-HCl buffer containing NaCl (100 mmol/L) at pH 7.4 and the BSA used as a substrate.The concentrations of plg, BSA and S-2444 were 1.5 μmol/L, 5 μmol/L and 4 nmol/L, respectively. They were prepared in a 96-well microplate. After the predetermined sample solutions (compounds FGFC1 and **F1**–**F7**) were added, pro-uPA (20 μmol/L) was added into the 96-well microplate. The microplate was then cultivated at 37 °C for 60 min. The continuous variation trend of absorbance was determined for evaluation of fibrinolytic activity on the slope of the plots of A405 nm within 150 min. In the blank group, an equal volume of Tris-HCl solution was used as a blank control. All sample solutions were prepared by adding a small amount of DMSO (<5%, *v*/*v*) solution to dissolve the derivatives.

### 3.4. Cytotoxicity, Expression of Fas/Apo-1 and IL-1 of Derivative F6 on HeLa Cells

#### 3.4.1. Cell Lines

HeLa (human cervical carcinoma) cells were incubated in medium (10% fetal bovine serum, 1% penicillin, 1% streptomycin) at 37 °C with 95% air and 5% CO_2_. Cells were passaged every 2–3 d and select exponential growth cells were used for further experiments.

#### 3.4.2. MTT Assay

The cell growth inhibitory activity of FGFC1 and derivative **F6** on HeLa cell lines were determined by the MTT (3-(4,5-dimethylthiazol-2yl)-2,5-diphenyl tetrazolium bromide) assay as we previously reported [41,42]. In addition, an equal volume of DMEM medium was added to the blank group.

#### 3.4.3. Fas/Apo-1 Assay on HeLa Cells

Fas/Apo-1 assay was carried out according to the manufacturer’s protocol [43,44,45]. A blank control group was set up. It was not added with the samples (FGFC1 and derivative **F6**) and the enzyme standard reagent, and the other steps were the same. The standard curve group and the sample group were tested. On the 96-well plate, the standard curve group was supplemented with 50 μL of the standard. The sample group tested was firstly added with a sample (FGFC1 and derivative **F6**) dilution of 40 μL, and then 10 μL of the sample was added (the final dilution of the sample was 5 times). After that, the plate was incubated at 37 °C for 30 min. Then, 50 μL chromogenic reagent A and 50 μL chromogenic reagent B per well were added, and the chromogenic reaction took place. Ultimately, the reaction was terminated by the addition of 50 μL stop solution per well and mixed thoroughly. After the addition of stop solution to reaction wells, the absorbance was recorded at OD 450 nm on an Enzyme-Labeled (microplate reader) Instrument after 15 min.

#### 3.4.4. IL-1 Assay on HeLa Cells

The cell culture method on the 96-well plate is in accordance with that in the MTT assay. The Elisa kit assay based on IL-1 of HeLa cells was consistent with the Fas/Apo-1 Elisa kit assay [46].

## 4. Conclusions

In conclusion, a series of new FGFC1 derivatives (**F1**–**F7**) were synthesized by chemical modification at C-2 and C-2′ phenol groups moieties and C-1″ carboxyl group. Derivatives **F1**–**F4** and **F6** displayed significant fibrinolytic activities with EC_50_ of 59.7, 87.1, 66.6, 82.8 and 42.3 μM respectively, via enhancement of urokinase activity. Among them, derivative **F6** presented the most remarkable fibrinolytic activity (2.72-fold than that of FGFC1). The evaluation data showed that derivative **F6** possessed moderate cytotoxicity and apoptotic effect on HeLa cells in the Fas/Apo-1 assay and did not cause obvious inflammation (statistical significance *p* > 0.1), making derivative **F6** a potential antithrombotic agent towards clinical application. Moreover, the results suggest a simple and efficient method to enhance/optimize the anti-thrombotic activity of FGFC-1 via “one-pot” hydrophobic modification.

## Figures and Tables

**Table 1:** Fibrinolytic activities of FGFC1 and derivatives **F1**–**F7** in vitro ^a^.

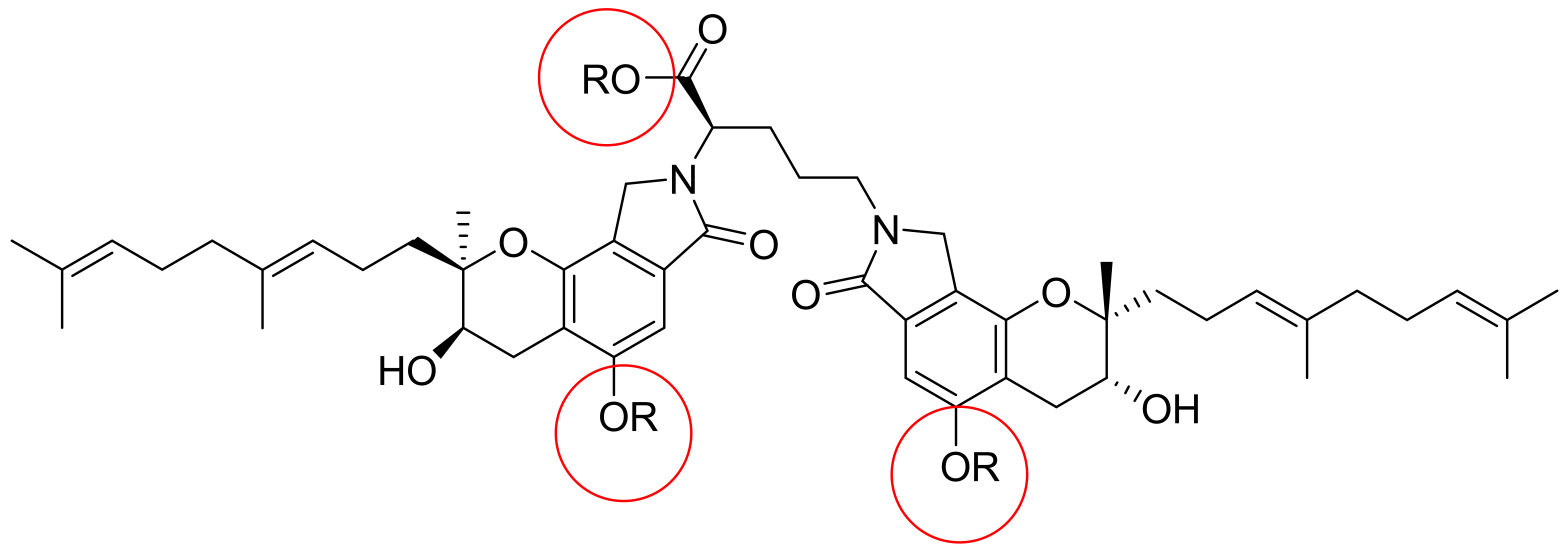		
**Compounds**	**R**	**EC50/μM**
**FGFC1**	H	115.0
**F1**	CH3	59.7
**F2**	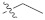	87.1
**F3**	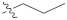	66.6
**F4**	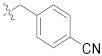	82.8
**F5**	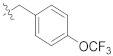	133.3
**F6**	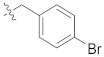	42.3
**F7**	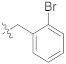	119.6

^a^ All values are the mean of two independent experiments.

## Data Availability

All data is contained within this article and Supplementary Materials.

## Supplementary Materials

The following are available online at https://www.mdpi.com/article/10.3390/md19040218/s1. Part 1: General procedure for preparation of derivatives F1–F7 [1,2]; Part 2: NRM Spectra.
