# Supplementary material for: Synthesis and Bioactivities of Marine Pyran-Isoindolone Derivatives as Potential Antithrombotic Agents
Source: Mar Drugs. 2021 Apr 15;19(4):218. doi: 10.3390/md19040218 (PMC8071544; doi:10.3390/md19040218)

Supplementary Information

Synthesis and Bioactivities of Marine Pyran-Isoindolone Derivatives as Potential Antithrombotic Agents

Yinan Wang [1,†], Hui Chen [2,†], Ruilong Sheng [3], Zhe Fu [1], **Junting Fan** [5], Wenhui Wu [1], Qidong Tu [4,*] and Ruihua Guo [1,6,7,*]

1 College of Food Science and Technology, Shanghai Ocean University, Shanghai 201306, China; wynshou@163.com (Y.W.); fzshou2019@163.com (Z.F.); whwu@shou.edu.cn (W.W.)
2 Shanghai Engineering Research Center of Hadal Science and Technology, College of Marine Sciences, Shanghai Ocean University, Shanghai 201306, China; h-chen@shou.edu.cn
3 CQM-Centro de Química da Madeira, Campus da Penteada, Universidade da Madeira, 9000-390 Funchal, Portugal; ruilong.sheng@staff.uma.pt
4 School of Pharmacy, Jiangxi Science and Technology Normal University, Nanchang 330013, China
5 School of Pharmacy, Nanjing Medical University, Nanjing 211166, China; juntingfan@njmu.edu.cn
6 Shanghai Engineering Research Center of Aquatic-Product Processing & Preservation, Shanghai 201306, China
7 Laboratory of Quality and Safety Risk Assessment for Aquatic Products on Storage and Preservation (Shanghai), Ministry of Agriculture, Shanghai 201306, China
* Correspondence: 1020100994@jxstnu.edu.cn (Q.T.); rhguo@shou.edu.cn (R.G.)
† These two authors contributed equally to this work.

1. General procedure for preparation of derivatives F1–F7[1,2]

Halogenated hydrocarbon or substituted benzyl (5.0 mmol, RI or RBr) was added drop wise to a solution of compound FGFC1 (50 mg, 0.057 mmol) and K_2CO_3 (79.5mg, 0.57 mmol) inacetone (2.0 mL). The reaction mixture was reacted at room temperature and the reaction was determined by TLC. The reaction mixture was diluted with 50 mL of CH_2Cl_2 and washed three times with 50 mL of brine. The organic layer was dried over anhydrous Na_2SO_4 and concentrated under reduced pressure. The residue was chromatographed on the silica gel to yield derivatives **F1–F7**.

1.1

Methyl(*R*)-5-((2*R*,3*R*)-2-((*E*)-4,8-dimethylnona-3,7-dien-1-yl)-3-hydroxy-5-methoxy-2-methyl -7-oxo-3,4,7,9-tetrahydropyrano[2,3-e]isoindol-8(2*H*)-yl)-2-((2*R*,3*R*)-2-((*E*)4,8dimethylnona-3,7-dien-1-y l)-3-hydroxy-5-methoxy-7-oxo-3,4,7,9-tetrahydropyrano[2,3-e]isoindol-8(2*H*)-yl)pentanoate(F1).

White amorphous powder, yield 62% (after chromatography with petroleum ether/ethyl acetate, 1:3); ^{1}H NMR ($CDCl_3$, 500 MHz) δ: 6.88 (1H, s), 6.83 (1H, s), 5.09–5.05 (4H, m), 4.41 (1H, d, *J* = 17.0 Hz), 4.24 (1H, d, *J* = 17.0 Hz), 4.205–4.208 (2H, m), 3.93 (2H, dd, *J* = 10.5, 5.0 Hz), 3.83 (3H, s), 3.82 (3H, s), 3.70 (3H, s), 3.59–3.53 (1H, m), 2.96–2.91 (2H, m), 2.76–2.68 (2H, m), 2.21–2.12 (6H, m), 2.06–2.02 (5H, m),

1.96–1.90 (5H, m), 1.66 (6H, S),1.62–1.61 (6H, m), 1.57 (6H, s), 1.36 (3H, s), 1.33 (3H, s).^{13}C NMR (125 MHz, $CDCl_3$) *δ*: 169.7, 168.5, 167.9, 156.6, 156.5, 147.5, 147.3, 135.0, 134.9, 134.7, 134.6, 133.3, 131.3, 128.7, 127.8, 123.1, 122.5, 121.3, 120.8, 111.6, 111.1, 96.7, 96.6, 78.3, 78.2, 68.5, 68.4, 66.5, 66.4, 65.2, 52.7, 46.5, 43.5, 40.9, 38.6 (2C), 36.1, 30.9, 28.7, 28.3, 26.2, 25.9, 25.6, 24.7, 24.4, 21.7, 20.6 (2C), 18.4, 18.1, 16.7 (2C), 15.1, 15.0. ESI-MS: *m/z* 911 ($C_{54}H_{74}N_2O_{10}$, $[M + H]^+$, Calc. 910.53).

1.2

Ethyl(*R*)-5-((2*R*,3*R*)-2-((*E*)-4,8-dimethylnona-3,7-dien-1-yl)-5-ethoxy-3-hydroxy-2-methyl-7-oxo-3,4,7,9-tetrahydropyrano[2,3-e]isoindol-8(2*H*)-yl)-2-((2*R*,3*R*)-2-((*E*)-4,8-dimethylnona-3,7-dien-1-yl)-5-ethoxy-3-hydroxy-7-oxo-3,4,7,9-tetrahydropyrano[2,3-e]isoindol-8(2*H*)yl)pentanoate

(F2).

White amorphous powder, yield 75% (after chromatography with petroleum ether/ethyl acetate, 1:2); ^{1}H NMR ($CDCl_3$, 500 MHz) *δ*: 6.88 (1H, s), 6.84 (1H, s), 5.10–5.05 (4H, m), 4.44 (1H, d, *J* = 16.5Hz), 4.22 (1H, d, *J* = 16.5Hz), 4.209–4.207 (2H, m), 4.06 (2H, t, *J* = 6.5Hz), 3.97–3.92 (7H, m), 3.72–3.67 (1H, m), 3.59–3.54 (1H, m), 3.00–2.96 (2H, m), 2.80–2.72 (2H, m), 2.18–2.11 (5H, m), 2.06–2.01 (5H, m), 1.96–1.89 (5H, m), 1.83–1.78 (5H, m), 1.66 (6H, s), 1.57 (12H, s), 1.36 (3H, s), 1.32 (3H, s), 1.05–1.02 (6H, m), 0.87 (3H, t, *J* = 7.5 Hz). ^{13}C NMR (125 MHz, $CDCl_3$) *δ*: 171.0, 169.6, 169.0, 158.2, 158.1, 148.3, 148.1, 135.6, 135.5, 132.2, 132.1, 131.3, 130.3, 124.1 (2C), 123.7, 123.6, 122.0, 121.3, 112.3, 112.0, 97.3, 97.2, 79.1, 79.0, 69.8 (2C), 67.5, 67.4, 66.8, 53.7, 47.5, 44.4, 41.9, 39.6 (2C), 37.1, 37.0, 27.3, 26.8, 26.6 (2C), 25.5 (2C), 25.4, 22.5, 21.8, 21.5, 19.2, 18.9, 17.6 (2C), 15.9 (2C), 10.5 (2C), 10.2. ESI-MS: *m/z* 953($C_{57}H_{80}N_2O_{10}$, $[M + H]^+$, Calc. 952.58).

1.3

Propyl(*R*)-5-((2*R*,3*R*)-2-((*E*)-4,8-dimethylnona-3,7-dien-1-yl)-3-hydroxy-2-methyl-7-oxo-5-propoxy-3,4,7,9-tetrahydropyrano[2,3-e]isoindol-8(2*H*)-yl)-2-((2*R*,3*R*)-2-((*E*)-4,8-dimethylnona-3,7-dien-1-yl)-3-hydroxy-7-oxo-5-propoxy-3,4,7,9-tetrahydropyrano[2,3-e]isoindol8(2*H*)yl)penta-noate(F3)

White amorphous powder, yield 70% (after chromatography with petroleum ether/ethyl acetate, 1:2); ^{1}H NMR ($CDCl_3$, 500 MHz) *δ*: 6.87 (1H, s), 6.84 (1H, s), 5.11–5.05 (4H, m), 4.46 (1H, d, *J* = 16.5 Hz), 4.23 (1H, d, *J* = 16.5Hz), 4.22–4.20 (2H,m), 4.18–4.14 (2H,m), 4.09–4.01 (4H,m), 3.94–3.92 (3H,m), 3.72–3.67 (1H, m), 3.59–3.54 (1H, m), 3.04–2.93 (2H, m), 2.80–2.72 (2H, m), 2.15–2.11 (8H, m), 2.06–2.02 (5H, m), 1.96–1.93(5H, m), 1.66 (12H, s), 1.63–1.61 (2H,m),1.57 (12H, s), 1.42–1.39 (6H, m), 1.36 (3H, s), 1.32 (3H, s), 1.23 (3H, t, *J*=7.5 Hz).^{13}C NMR (125 MHz, $CDCl_3$) *δ*: 170.9, 169.6, 169.0, 158.1, 158.0, 148.3, 148.1, 135.7, 135.6, 132.1, 131.4, 131.33, 131.32, 124.2, 123.6, 122.1, 121.3, 119.7, 119.5, 111.8, 111.8, 97.3, 97.2, 79.1, 79.0, 67.6,

67.5, 64.0, 63.9, 61.3, 53.6, 47.5, 44.4, 41.9, 39.6 (2C), 37.1, 37.0, 27.3, 26.8 (2C), 26.6 (2C), 25.6 (2C), 25.5, 22.5 (2C), 21.6 (2C), 20.1, 19.3, 19.0, 17.6 (2C), 15.9, 15.8, 14.7 (2C), 14.1. ESI-MS: *m/z* 1017 ($C_{60}H_{86}N_2O_{10}$, [M + Na]$^+$, Calc. 994.63).

1.4

Cyanobenzyl (*R*)-2,5-Bis((2*R*,3*R*)-5-((4-cyanobenzyl)oxy)-2-((*E*)-4,8-dimethylnona-3,7-dien-1-yl)-3 hydroxy-2-methyl-7-oxo-3,4,7,9tetrahydropyrano[2,3-e]isoindol-8(2*H*)yl)pentanoate(F4)

White amorphous powder, yield 56 %(after chromatography with petroleum ether/ethyl acetate,); ^{1}H NMR ($CDCl_3$, 500 MHz) δ:7.62 (2H, d, *J* = 7.5 Hz), 7.59 (2H, d, *J* = 7.5 Hz), 7.55 (3H, d, *J* = 7.5 Hz), 7.51 (3H, d, *J* = 7.5 Hz), 7.46 (2H, d, *J* = 7.5 Hz),6.94 (1H, s), 6.90 (1H, s), 5.17–5.05 (6H,m),4.74(4H, s), 4.40 (1H, d, J = 16.5 Hz), 4.27–4.18 (3H, m), 3.98–3.96 (2H, m), 3.78–3.45 (3H, m),3.07–3.03 (2H, m), 2.87–2.81 (2H, m), 2.21–2.14 (8H, m), 2.04–2.01 (6H, m), 1.96–1.95 (6H, m),1.65(6H,s),1.57 (12H, s),1.26(6H, s).^{13}C NMR (125 MHz, $CDCl_3$) *δ*: 170.6, 169.5, 168.8, 157.4, 157.3, 148.6, 148.4, 138.3, 138.2, 136.8, 135.9, 135.8, 132.1, 131.9, 131.6, 131.5, 131.3, 131.0, 130.9, 130.4, 130.3, 130.1, 129.9, 129.7, 129.4, 129.2 (2C), 124.1, 123.4, 123.3, 122.9, 122.3, 118.8 (2C), 118.6 (2C), 118.3 (2C), 112.7, 112.4 (2C), 112.1, 97.5, 97.4, 79.4, 79.3, 68.9, 68.8, 67.4, 65.6, 63.9, 53.7, 47.6, 44.7, 41.8, 39.6, 37.0, 35.9, 31.9, 29.6, 29.1, 27.2, 27.1, 26.9, 26.6, 25.7, 25.4, 22.7, 21.6, 19.4, 19.2, 17.6 (2C), 16.0, 14.1. ESI-MS: *m/z* 1214 ($C_{75}H_{83}N_5O_{10}$, [M + H]$^+$, Calc. 1213.61).

1.5

4-(Trifluoromethoxy)benzyl(*R*)-2,5-bis((2*R*,3*R*)-2-((*E*)-4,8-dimethylnona-3,7-dien-1-yl)-3-hydroxy-2-methyl-7-oxo-5-((4-(trifluoromethoxy)benzyl)oxy)-3,4,7,9-tetrahydropyrano[2,3-e]isoindol-8(2H)-yl) pentanoate(F5)

White amorphous powder, yield 72.6 %(after chromatography with petroleum ether/ethyl acetate); ^{1}H NMR ($CDCl_3$, 500 MHz) δ:7.47–7.45 (4H, m), 7.32 (2H, d, *J*=8.5 Hz),7.24–7.22 (4H,m), 7.16–7.12(2H, m), 6.96 (1H, s), 6.94 (1H, s),5.14 (2H, s),5.09–5.05 (8H, m), 4.42 (1H, d, *J* = 16.5Hz), 4.27–4.20 (3H, m), 3.96–3.93 (2H, m),3.73–3.69 (1H, m),3.55–3.52 (2H, m),3.05–3.01(2H, m),2.85–2.77(2H,m),2.23–2.11 (6H, m), 2.06–1.99 (6H, m), 1.97–1.93 (6H, m),1.65(6H,s),1.64–1.62 (2H, m),1.57 (12H, s),1.42 (3H, s),1.37 (3H, s).^{13}C NMR (125 MHz, $CDCl_3$) *δ*: 170.8, 169.4, 168.8, 157.7, 157.6, 149.1, 148.9, 147.1, 139.2, 138.6, 136.0, 135.9, 135.4, 134.1, 132.5, 131.5, 129.6 (2C), 128.7 (2C), 128.1, 127.9, 127.8, 127.3, 127.2, 124.5, 124.4, 124.2, 124.1, 124.0 (2C), 123.5 (2C), 122.9, 122.8, 122.3, 121.1 (2C), 119.1 (2C), 112.5, 112.0, 97.7, 97.6, 79.3, 79.2, 69.5, 69.4, 67.6, 67.4, 66.0, 53.7, 47.5, 44.5, 41.9, 39.7, 37.1, 34.8, 34.5, 29.7 (2C), 29.4, 27.0, 26.6 (2C), 22.7 (2C), 21.6, 21.1, 19.4, 19.1, 17.6 (2C), 16.0, 14.1. ESI-MS: *m/z* 1343 ($C_{75}H_{83}F_9N_2O_{10}$, [M + H]$^+$, Calc. 1342.59).

1.6

4-Bromobenzyl(*R*)-2,5-bis((2*R*,3*R*)-5-((4-bromobenzyl)oxy)-2-((*E*)-4,8-dimethylnona-3,7-dien-1-yl)-3-hydroxy-2-methyl-7-oxo-3,4,7,9-tetrahydropyrano[2,3-e]isoindol-8(2*H*)yl)pentanoate(F6)

White amorphous powder, yield 82.3%(after chromatography with petroleum ether/ethyl acetate); ^{1}H NMR ($CDCl_3$, 500 MHz) δ:7.50-7.48 (4H, m), 7.40 (2H, d, *J*=8.5Hz), 7.30-7.28 (4H, m), 7.14 (2H, d, *J*=8.5Hz), 6.93 (1H, s), 6.90 (1H, s), 5.13–5.01 (10H, m),4.40 (1H, d, *J*=17.0Hz),4.25–4.15(3H, m),3.95–3.91 (2H, m),3.71–3.65(1H, m),3.55–3.51(2H, m),3.04–2.99 (2H,m),2.85–2.77(2H,m),2.20–2.10 (7H, m), 2.05–2.00 (5H, m), 1.97–1.91 (6H, m),1.65(6H,s),1.63–1.61 (2H,m), 1.56(12H, s),1.37(3H, s),1.34(3H, s).^{13}C NMR (125 MHz, $CDCl_3$) δ: 170.1, 169.5, 168.8, 157.6, 157.5, 148.4, 148.3, 135.9, 135.8, 135.6, 135.5, 134.3, 132.2, 131.7 (2C), 131.59, 131.6, 131.4, 131.3, 131.1, 130.4, 130.3, 129.7, 129.1, 128.8, 128.5, 124.1, 124.0, 123.5, 123.4, 122.7, 122.3, 122.0, 121.9, 121.8, 119.0, 118.9, 112.5, 112.1, 97.6, 97.5, 79.3, 79.2, 69.4, 69.3, 67.4, 67.3, 66.1, 53.6, 47.5, 44.5, 41.8, 39.6, 37.0, 33.4, 31.9, 29.7, 29.3, 27.1, 26.9, 26.6, 25.7, 25.4, 23.2, 22.7, 21.6, 19.4, 19.1, 17.6 (2C), 16.0, 14.1. ESI-MS: *m/z* 1373 ($C_{72}H_{83}Br_3N_2O_{10}$, $[M + H]^+$, Calc. 1372.36).

1.7

2-bromobenzyl(*R*)-2,5-bis ((2*R*,3*R*)-5-((2-Bromobenzyl)oxy)-2-((*E*)-4,8-dimethylnona-3,7-dien-1-yl)-3-hyd-roxy-2-methyl-7-oxo-3,4,7,9-tetrahydropyrano[2,3-e]isoindol-8(2*H*)-yl)pentanoa-te(F-7).

White amorphous powder, yield 73.2% (after chromatography with petroleum ether/ethyl acetate); ^{1}H NMR ($CDCl_3$, 500 MHz) δ:7.58–7.55 (4H, m),7.51 (1H, d,*J*=8.0Hz), 7.34–7.32 (3H, m), 7.25 (1H, t, *J*=8.0 Hz), 7.17–7.13 (3H,m), 7.01 (1H,s), 6.98 (1H, s), 5.22–5.05 (10H, m), 4.48 (1H, d, *J* = 16.5Hz), 4.29–4.18 (3H, m), 3.96–3.95 (2H, m), 3.75–3.47 (3H, m),3.10–3.06 (2H, m), 2.91–2.83 (2H, m), 2.22–2.13(7H, m),2.05–2.01 (5H,m), 1.97–1.93 (6H, m),1.65(6H,s),1.62–1.60 (2H, m),1.57 (12H, s),1.38 (3H, s),1.34 (3H, s).^{13}C NMR (125 MHz, $CDCl_3$) δ: 170.6, 169.5, 168.8, 157.49, 157.50, 148.4, 148.3, 136.0, 135.91, 135.93, 135.8, 134.6, 132.8 (2C), 132.6, 132.4, 131.4, 131.3, 130.1, 129.9, 129.8 (2C), 129.3, 129.2, 128.6, 127.5, 127.4, 124.2, 124.1, 123.5, 123.4, 123.3, 122.9, 122.3, 122.1, 119.1, 119.0, 112.5, 112.1, 97.8, 97.7, 79.3, 79.2, 69.8, 69.7, 67.5, 67.4, 66.6, 53.6, 47.5, 44.5, 41.9, 39.6 (2C), 37.1, 37.0, 29.7, 29.3, 27.3, 27.2, 26.9, 26.6, 25.7, 25.5, 22.7, 21.6, 19.4, 19.1, 17.7 (2C), 16.0, 14.1. ESI-MS: *m/z* 1373 ($C_{72}H_{83}Br_3N_2O_{10}$, $[M + H]^+$, Calc. 1372.36).

References:

[1] Amanda, S.R.; Gabriel, N.V.; Sergio, H.F.; María, Y.R.; Maximiliano, I.B.; Samuel, E.S. Semisynthesis, ex vivo evaluation, and SAR studies of coumarin derivatives as potential

antiasthmatic drugs. *Eur. J. Med. Chem*. **2014**, *77*, 400-408.
[2] Jane, A.P. Cell sensitivity assays: The MTT assay. *Methods. In. Mol. Med*. 1999, 80.

2.NMR Spectra

2.1 F1 NMR Spectra

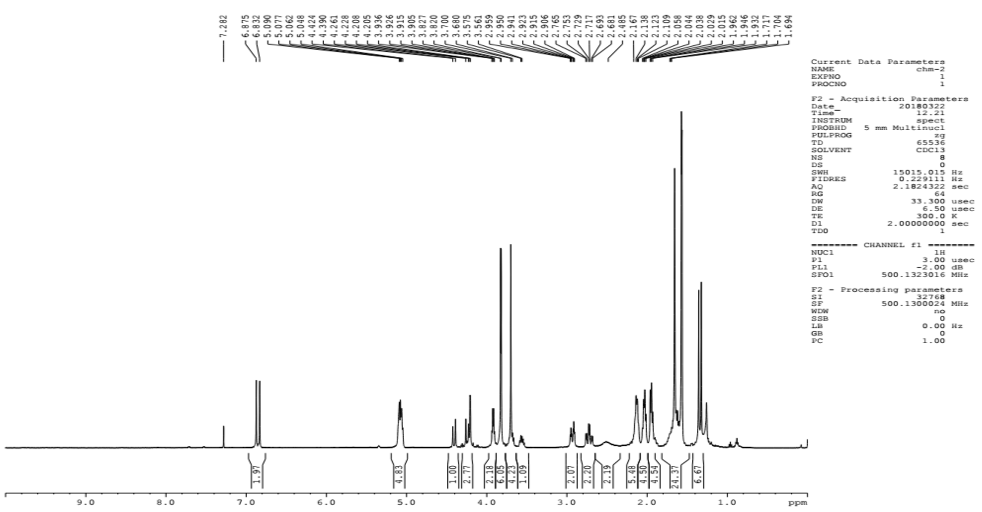

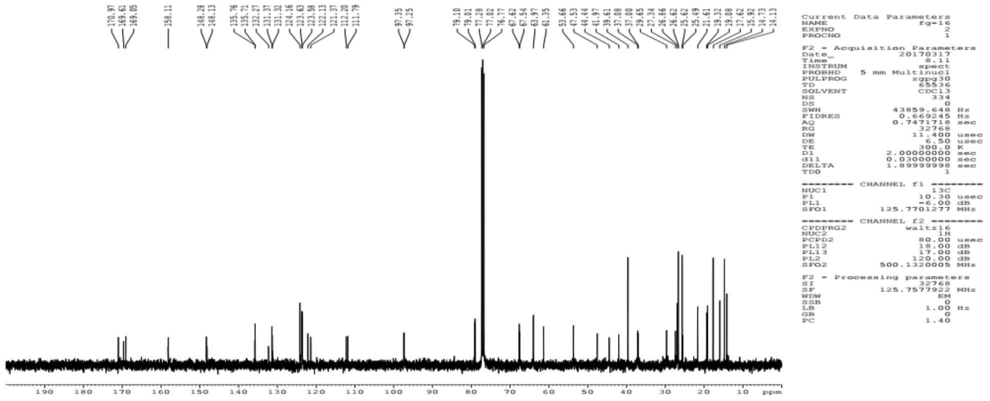

2.2 F2 NMR Spectra

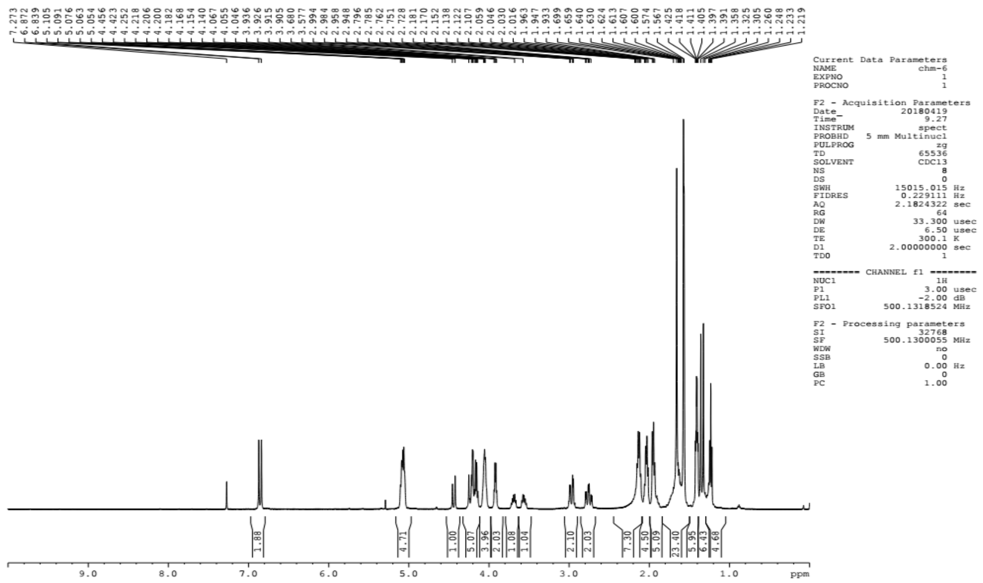

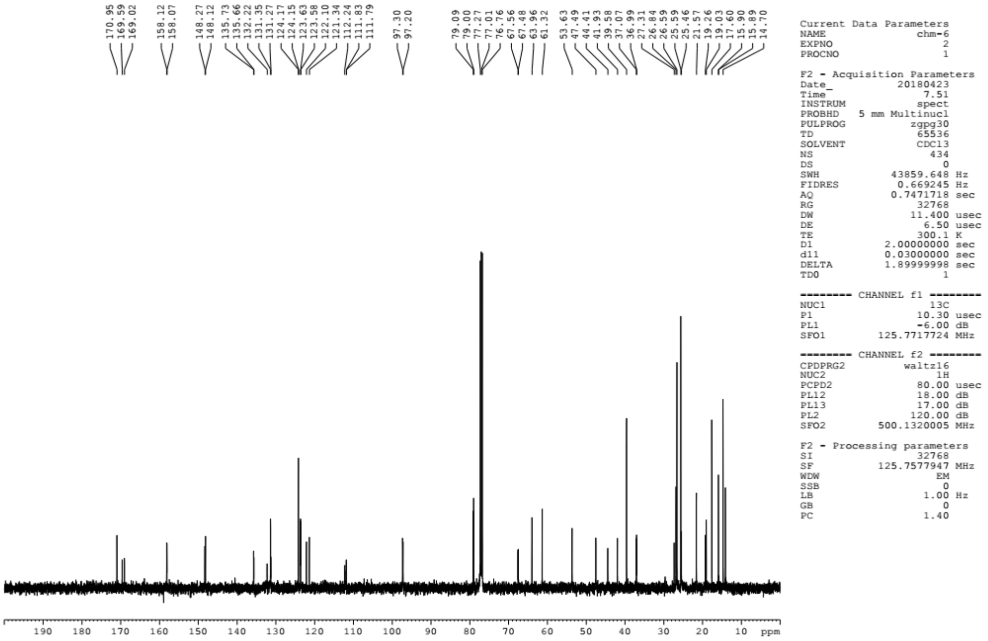

2.3 **F3** NMR Spectra

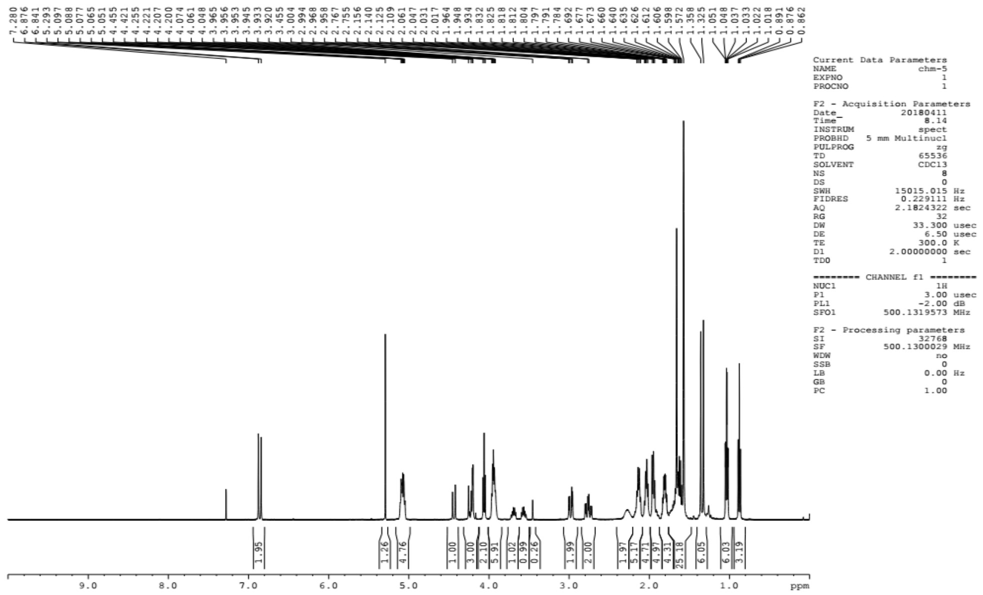

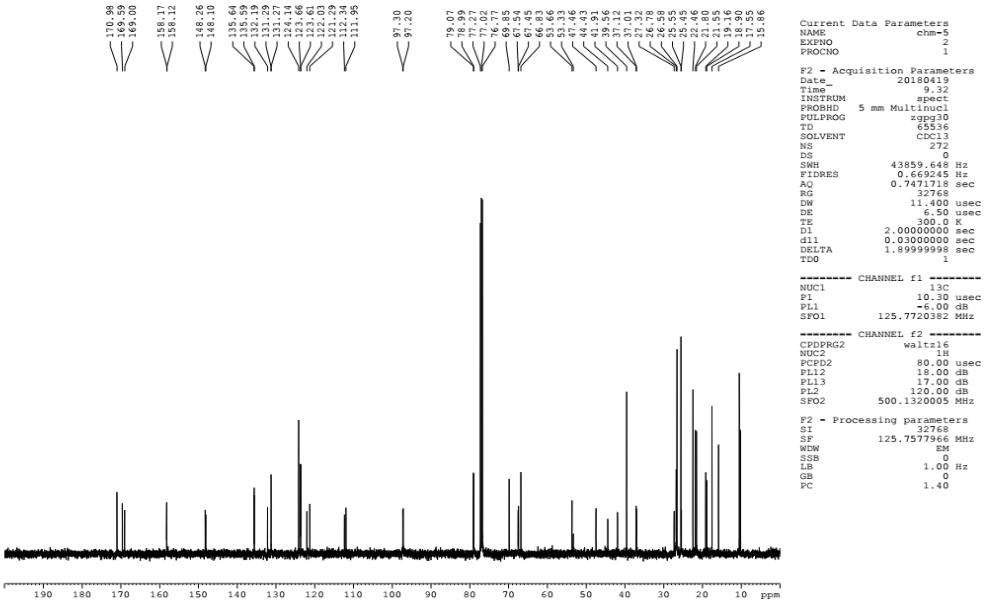

2.4 F4 NMR Spectra

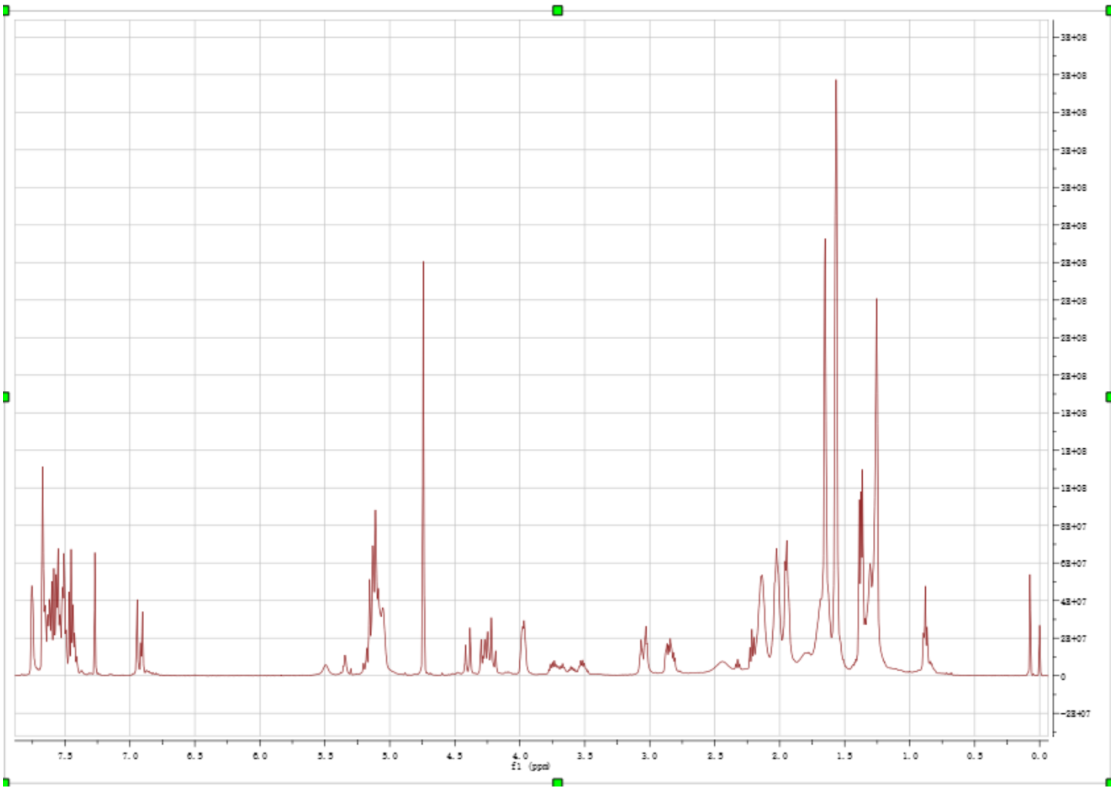

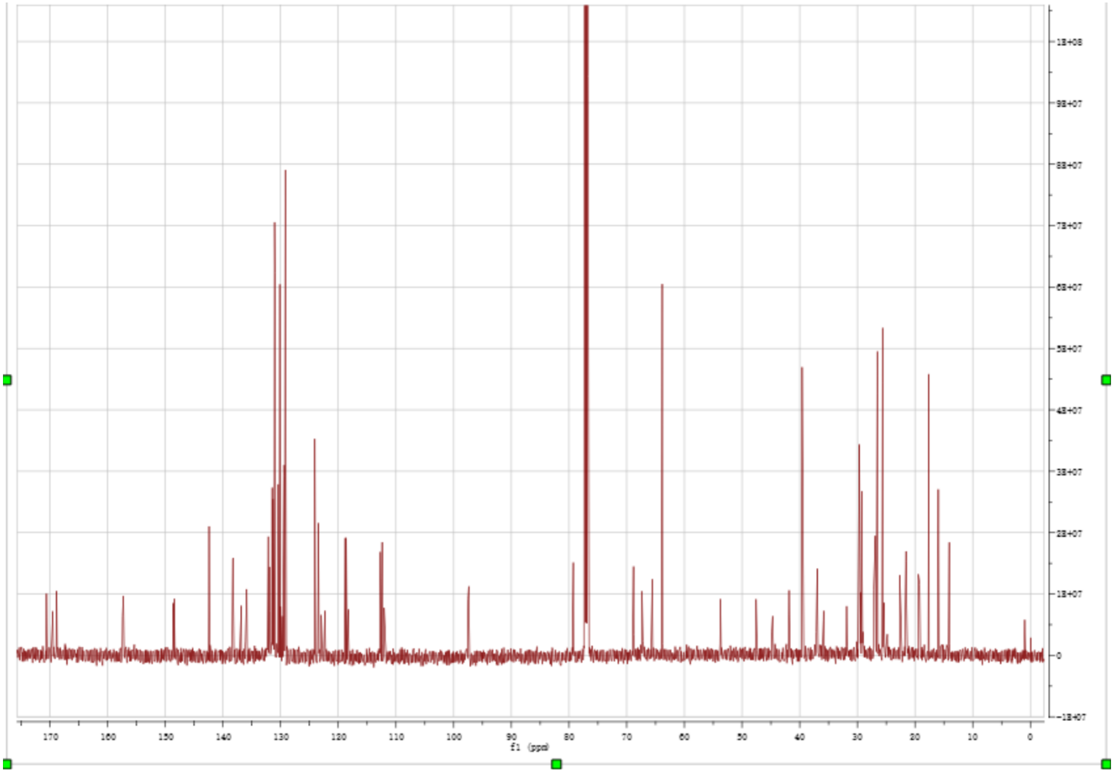

2.5 F5 NMR Spectra

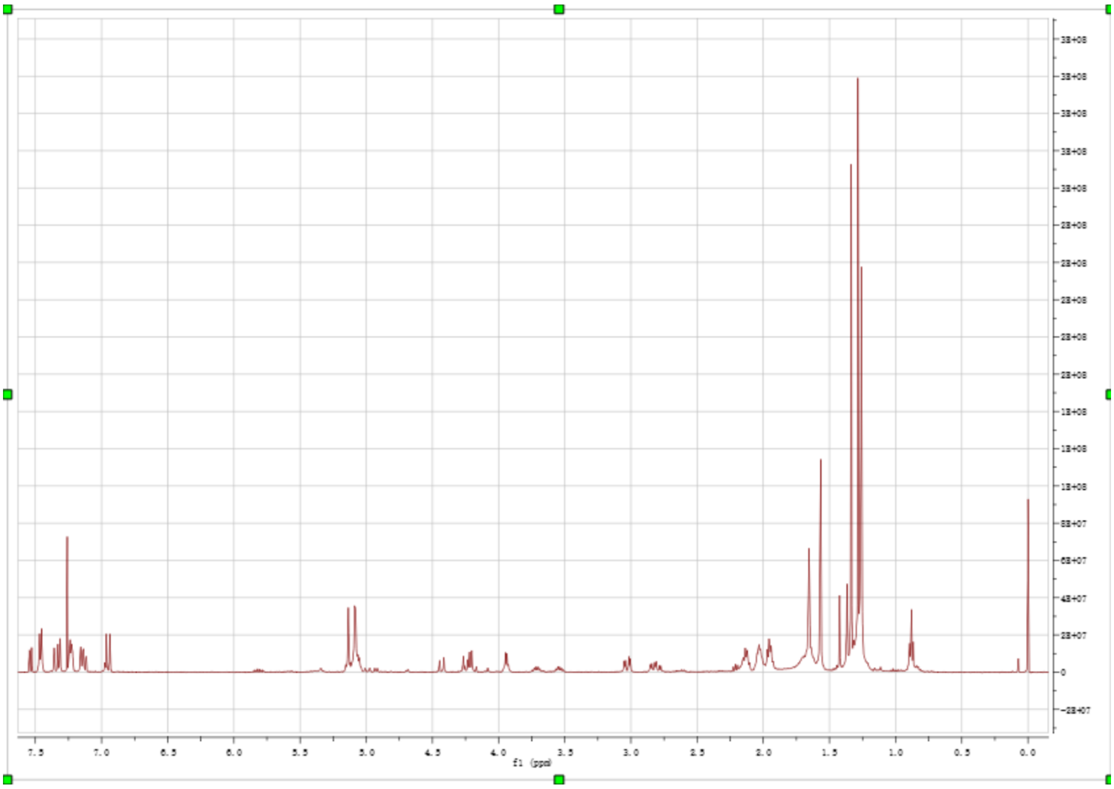

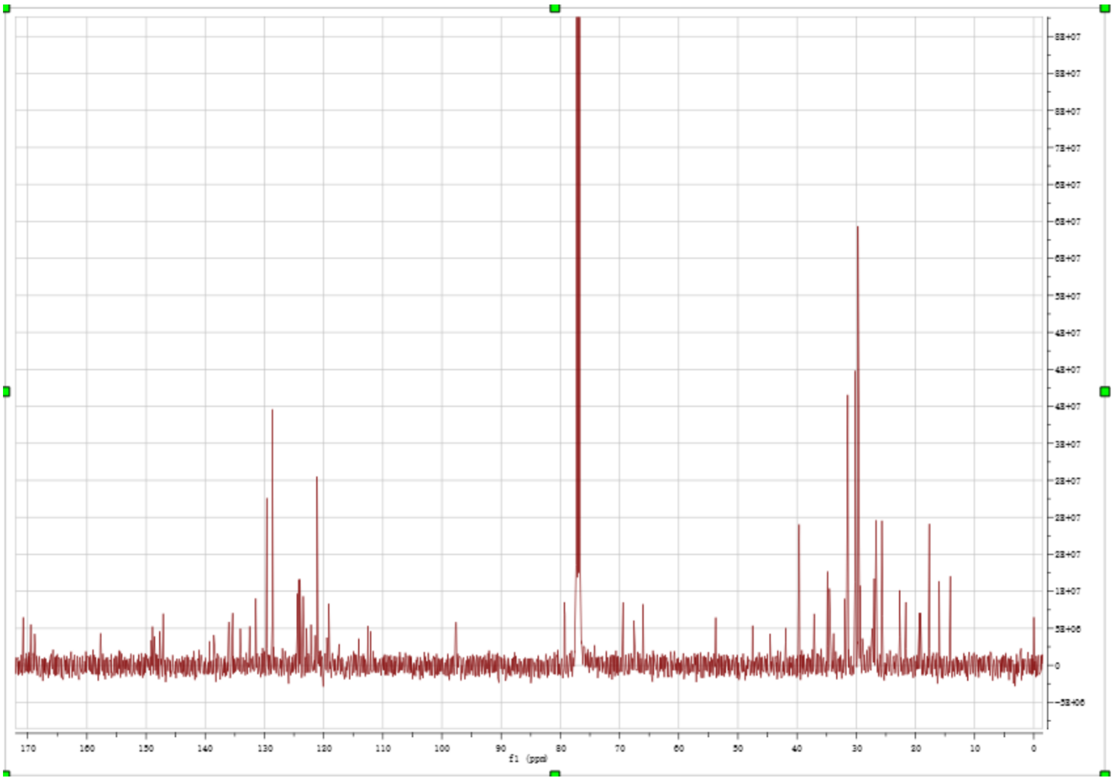

2.6 F6 NMR Spectra

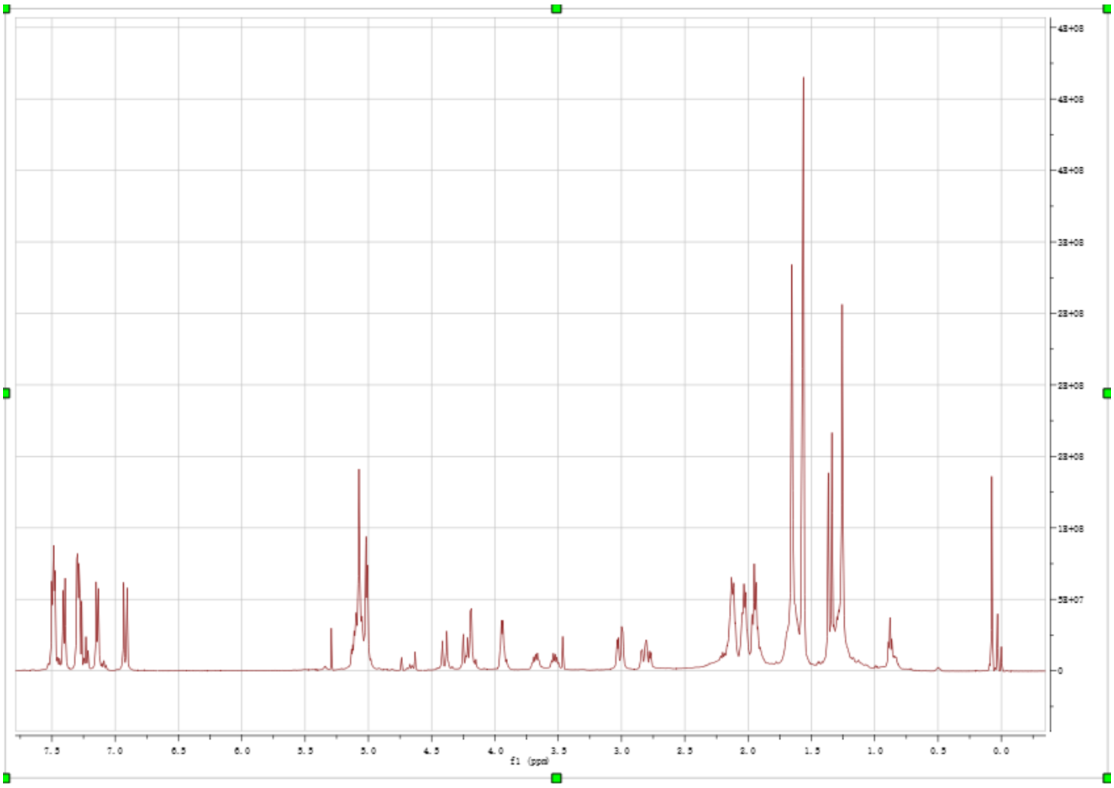

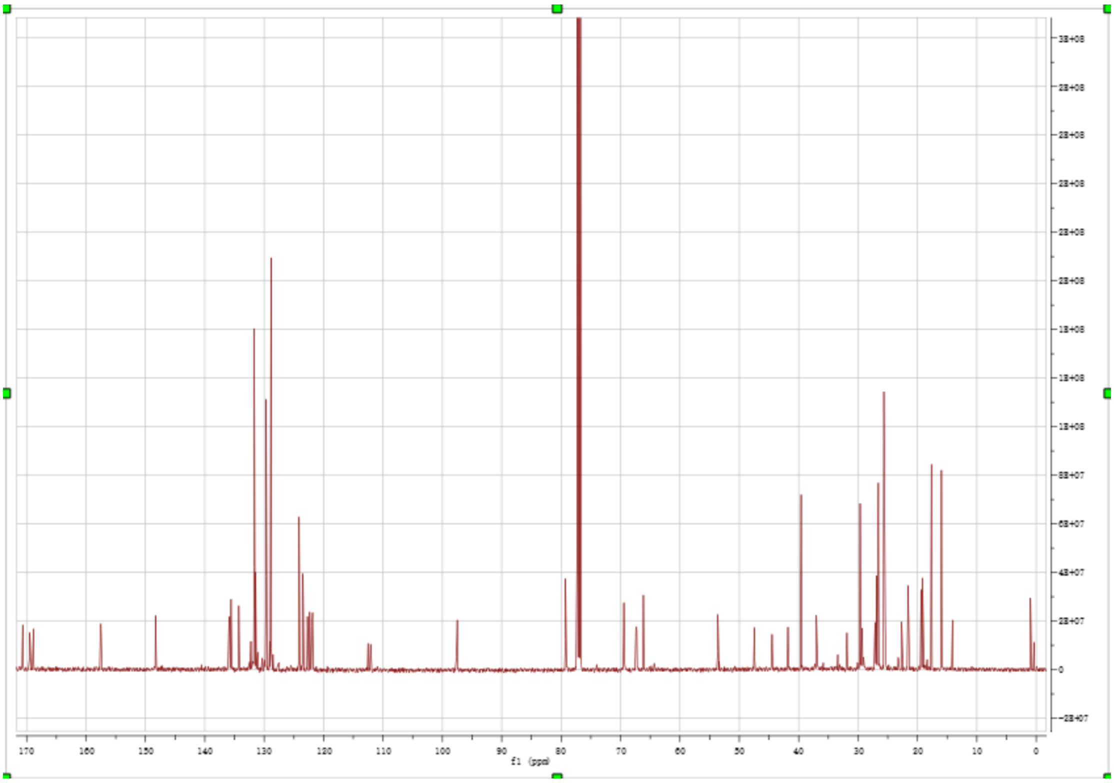

2.7 F7 NMR Spectra

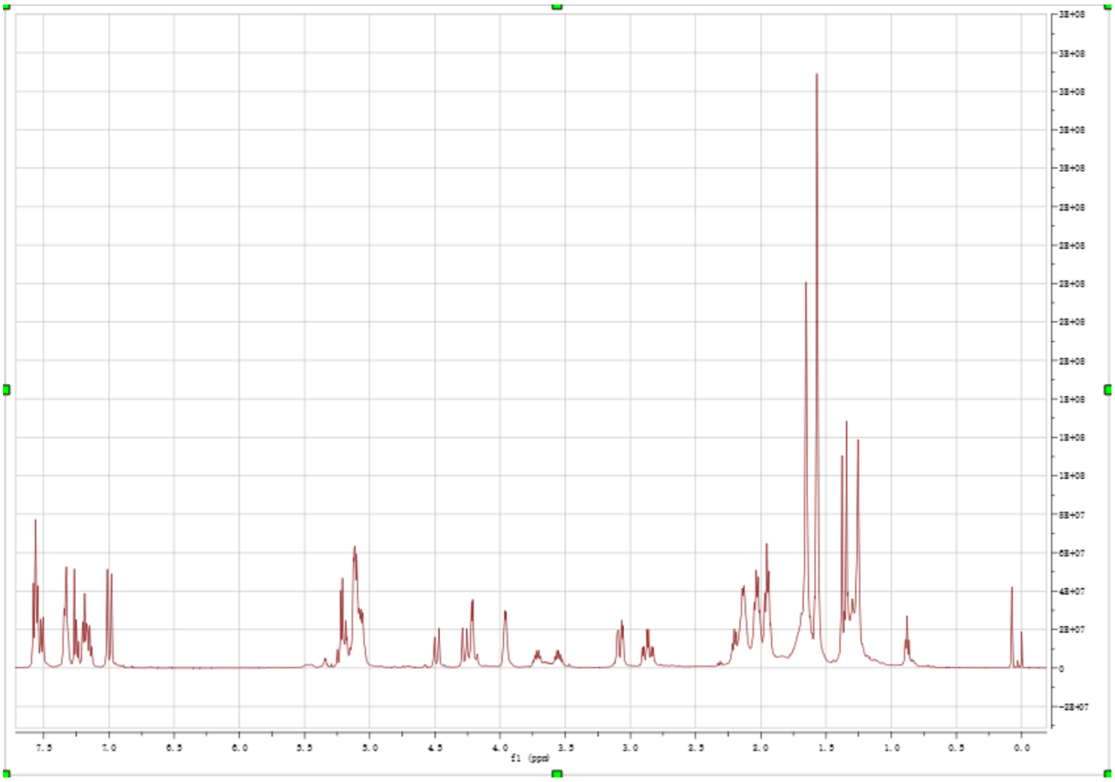

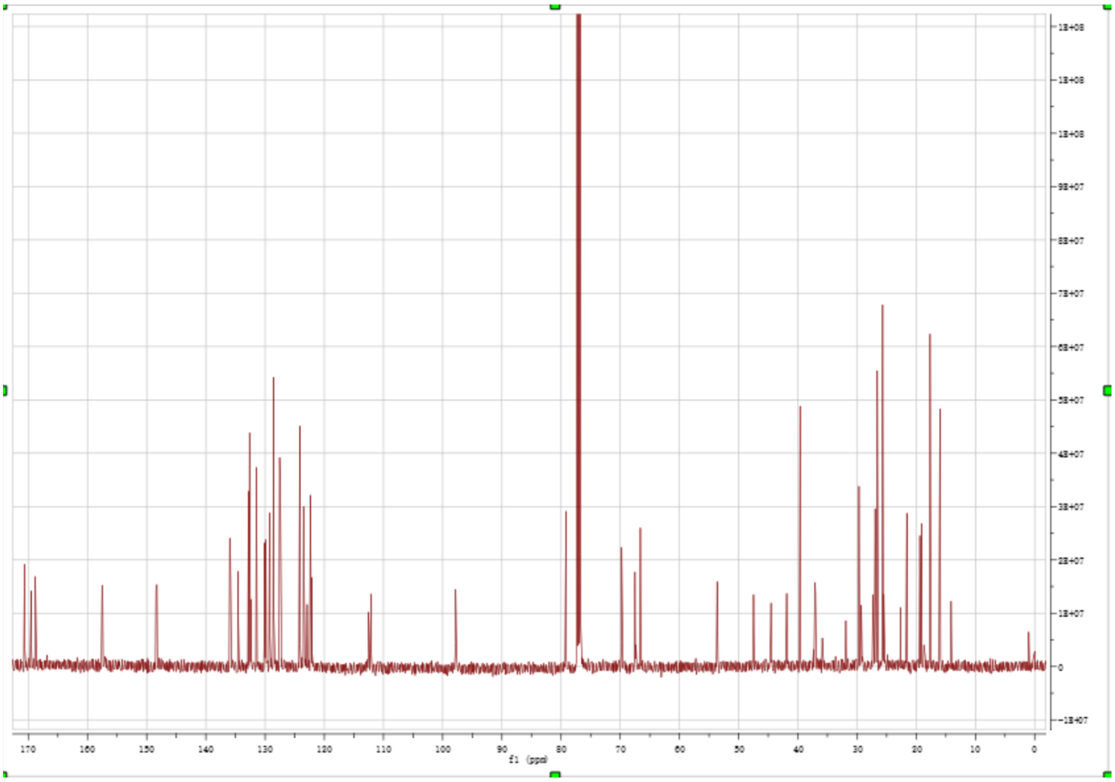

Supplement: Supplementary file 1 [file marinedrugs-19-00218-s001.zip › marinedrugs-1164662-supplementary.pdf]
